# Electrical Impedance Tomography during Abdominal Laparoscopic Surgery: A Physiological Pilot Study

**DOI:** 10.3390/jcm12237467

**Published:** 2023-12-01

**Authors:** Michela Rauseo, Savino Spadaro, Lucia Mirabella, Antonella Cotoia, Donato Laforgia, Gennaro Gaudino, Francesca Vinella, Giuseppe Ferrara, Adriana Gattullo, Livio Tullo, Gilda Cinnella

**Affiliations:** 1 Department of Surgical and Medical Science, Anesthesia and Intensive Care Unit, Policlinico Riuniti di Foggia, University of Foggia, Viale Pinto 1, 71122 Foggia, Italy; lucia.mirabella@unifg.it (L.M.); antonella.cotoia@unifg.it (A.C.); laforgia.don@gmail.com (D.L.); gennarogaudino1991@gmail.com (G.G.); francesca.vinella@hotmail.com (F.V.); giuseppeferrara89@gmail.com (G.F.); adriana.gattullo@gmail.com (A.G.); liviotullo@yahoo.it (L.T.); gilda.cinnella@unifg.it (G.C.); 2 Department of Translational Medicine, Anesthesia and Intensive Care Unit, Azienda Ospedaliera Universitaria di Ferrara, University of Ferrara, Via Aldo Moro 8, 44124 Ferrara, Italy; savinospadaro@gmail.com

**Keywords:** mechanical ventilation, laparoscopy, recruitment maneuver, positive end-expiratory pressure, electrical impedance tomography, anesthesia, pneumoperitoneum, driving pressure

## Abstract

Background: Both general anesthesia and pneumoperitoneum insufflation during abdominal laparoscopic surgery can lead to atelectasis and impairment in oxygenation. Setting an appropriate level of external PEEP could reduce the occurrence of atelectasis and induce an improvement in gas exchange. However, in clinical practice, it is common to use a fixed PEEP level (i.e., 5 cmH_2_O), irrespective of the dynamic respiratory mechanics. We hypothesized setting a PEEP level guided by EIT in order to obtain an improvement in oxygenation and respiratory system compliance in lung-healthy patients than can benefit a personalized approach. Methods: Twelve consecutive patients scheduled for abdominal laparoscopic surgery were enrolled in this prospective study. The EIT Timpel Enlight 1800 was applied to each patient and a dedicated pneumotachograph and a spirometer flow sensor, integrated with EIT, constantly recorded respiratory mechanics. Gas exchange, respiratory mechanics and hemodynamics were recorded at five time points: T0, baseline; T1, after induction; T2, after pneumoperitoneum insufflation; T3, after a recruitment maneuver; and T4, at the end of surgery after desufflation. Results: A titrated mean PEEP of 8 cmH_2_O applied after a recruitment maneuver was successfully associated with the “best” compliance (58.4 ± 5.43 mL/cmH_2_O), with a low percentage of collapse (10%), an acceptable level of hyperdistention (0.02%). Pneumoperitoneum insufflation worsened respiratory system compliance, plateau pressure, and driving pressure, which significantly improved after the application of the recruitment maneuver and appropriate PEEP. PaO_2_ increased from 78.1 ± 9.49 mmHg at T0 to 188 ± 66.7 mmHg at T4 (*p* < 0.01). Other respiratory parameters remained stable after abdominal desufflation. Hemodynamic parameters remained unchanged throughout the study. Conclusions: EIT, used as a non-invasive intra-operative monitor, enables the rapid assessment of lung volume and regional ventilation changes in patients undergoing laparoscopic surgery and helps to identify the “optimal” PEEP level in the operating theatre, improving ventilation strategies.

## 1. Introduction

The induction of general anesthesia and the shift from spontaneous breathing to controlled mechanical ventilation may lead the alveoli to collapse [1,2], being associated with a significant reduction in functional residual capacity (FRC) and the formation of atelectasis [3]. This is responsible for the increased risk of intraoperative hypoxia and postoperative pulmonary complications (PPCs) [4].

Abdominal laparoscopic surgery could lead to the development of further atelectasis due to an increase in intraabdominal pressure (IAP) determined by cranial diaphragm movement [5]. During laparoscopic abdominal surgery, pulmonary ventilation is further impaired by the application of pneumoperitoneum insufflation [5]. The use of recruitment maneuvers and positive end-expiratory pressure (PEEP) is therefore recommended during laparoscopic surgery [6] to prevent atelectasis and keep the alveoli open in agreement with the “open lung” concept [7,8,9].

Recruitment maneuvers and positive end-expiratory pressure (PEEP) might reduce the risk of atelectasis [10,11], but an inappropriate higher PEEP level could also lead to overdistension in the non-dependent areas. Currently, several studies have demonstrated that mechanical ventilation with low tidal volume (Vt) ventilation combined with the application of an external PEEP might be beneficial not only in injured lungs but in healthy lungs as well [12,13,14]. However, even if PEEP is widely used in clinical practice, it remains a matter of debate as to how to individually titrate the adequate PEEP level for patients undergoing laparoscopic abdominal surgery.

Electrical impedance tomography (EIT) is well known as a non-invasive functional imaging tool able to detect dynamically regional changes in ventilation during mechanical ventilation [14,15]. Several studies have shown that EIT measurements are currently feasible in the peri-operative period, improving ventilation strategies during surgical procedures and preventing pulmonary complications or the alteration of respiratory parameters [13,14,15,16,17].

We thus hypothesized that an EIT-guided PEEP level in lung-healthy patients undergoing abdominal laparoscopic surgery would improve oxygenation and respiratory system compliance at the end of surgery, after the application of a recruitment maneuver, avoiding the dangerous effect of ventilation caused by the impact of general anesthesia on dorsal lung regions or pneumoperitoneum insufflation and the application of a “blinded” external PEEP level.

## 2. Methods

### 2.1. Patient Selection

After receiving the approval of the local ethic committee (129/CE/2018, 18 September 2018) and obtaining written informed consent, all consecutive adults patients scheduled for abdominal laparoscopic surgery were enrolled. The study was carried out in the Department of General Surgery at Policlinico Riuniti University Hospital of Foggia by the team of anesthesiologists working in the OR from January to June 2020. The inclusion criterion was patients aged 18 years or older scheduled for laparoscopic abdominal surgery (rectum and colon, performed by the same surgeons) undergoing general anesthesia. The exclusion criteria were as follows: surgical conversion to laparotomy; individuals older than 80 years; severe cardiac or pulmonary comorbidities or another disorder that might have compromised the safety of the trial procedure; a body mass index higher than 40 kg/m^2^; the presence of pregnancy, tracheostomy, facial, neck, or chest wall abnormalities; a history of abdominal aortic aneurysm surgery, chemotherapy, or immunosuppressive therapy within 3 months; and individuals unwilling or unable to provide informed consent. This study design conformed to the CONSORT guidelines.

### 2.2. Anesthesia Management

Following preoxygenation with FiO_2_ 0.8 for 3 min, anesthesia was induced with 2–3 mg of midazolam, fentanyl at a dose of 2 mcg/kg ideal body weight (IBW), propofol at a dose of up to 2 mcg/kg total body weight (TBW) titrated to loss of eyelash reflex, and rocuronium at a dose of 0.6 mg/kg IBW. Anesthesia was maintained with sevoflurane administered using the low-flow technique with end-tidal (etSevo) concentrations of 0.7–1 MAC and additional fentanyl up to a total dose of 5–6 mcg/kg IBW depending on the type of surgery. Neuromuscular blockade was monitored using the train of four (TOF) technique and sugammadex was administered for block reversal upon the completion of surgery.

### 2.3. Respiratory Mechanics and Ventilation Setting

After intubation, patients were ventilated with a tidal volume (Vt) of 6–8 mL/kg PBW with FiO_2_ 0.4–0.5 with a minimum PEEP value of 5 cmH_2_O. The respiratory rate was adjusted to keep end-tidal carbon dioxide (etCO_2_) between 35 and 45 mmHg. Additionally, in every patient, a recruitment maneuver performed with a decremental trial followed by an incremental trial [11], until reaching a peak inspiratory pressure of 40 cmH_2_O.

Static respiratory system compliance (Cs), plateau pressure (Pplat), driving pressure (Pdrive), etCO_2_ and SpO_2_ values, respiratory rate (RR), mean arterial pressure (MAP), and heart rate (HR) were obtained from the anesthetic workstation (Datex-Ohmeda Aespire or Aestiva). If SpO_2_ values fell to ≤92% during anesthesia, FiO_2_ was increased to 0.8. In order to monitor the regional ventilation distribution and evaluate the direct effects of the recruitment maneuver, a 32-electrode belt of an appropriate size was selected to match chest circumference at the level of the 4th intercostal space. The belt was subsequently connected to electrical impedance tomography (Enlight 1800-Timpel, Sao Paulo, Brazil). After high signal quality was confirmed, monitoring was initiated and only interrupted during electrocautery by disconnecting the electrode belt from the impedance tomograph. If signal quality was low, the measurement was restarted. In cases where a high signal quality was not obtained or was lost during anesthesia and could not be restored before the next data collection time point, the patient was excluded from the study. Respiratory mechanics were constantly recorded using a pneumotachograph and a spirometer flow sensor, connected to the same monitor machine that displayed the changes in ventilation distribution. All parameters were recorded at 5 different time points: before induction of anesthesia in a spontaneously breathing patient in a ramp position (T0); 5 min after intubation and induction of anesthesia (T1); 5 min after pneumoperitoneum insufflation with 12 mmHg(T2); 5 min after the application of a recruitment maneuver (T3); and 5 min after desufflation of the pneumoperitoneum at the end of the surgery (T4).

### 2.4. Sample Size and Statistics

A sample size calculation was performed using data from our previous study based on the effects of recruiting maneuvers in patients undergoing cholecystectomy laparoscopic surgery. Based on these data, a mean PEEP of 8 cmH_2_O with an SD of 1 was considered the “best” applied. By using a one-sample, one-sided test, the calculated sample size was 9 patients; this number was increased to 12 to allow for an expected drop-out of around one-third of the patients and was used for patient enrolment. The α and β errors for the sample size were chosen as 0.05 and 95%, respectively.

The statistical comparison of demographic factors, respiratory mechanics, and hemodynamic and gas exchange data was performed between the four study steps. Non-continuous data are expressed as numbers and percentages. Continuous data were tested for normal distribution using the Shapiro–Wilk test and are presented as means and standard deviations.

Data analysis was performed by means of repeated-measure ANOVA. If significant, the Kruskal–Wallis test was applied for post hoc comparison between the different study steps. A *p* value less than 0.05 was considered statistically significant. Statistical analysis was performed using Jamovi v. 1.2.27.0 (www.jamovi.org, accessed on 1 July 2020).

## 3. Results

**Figure 1:**
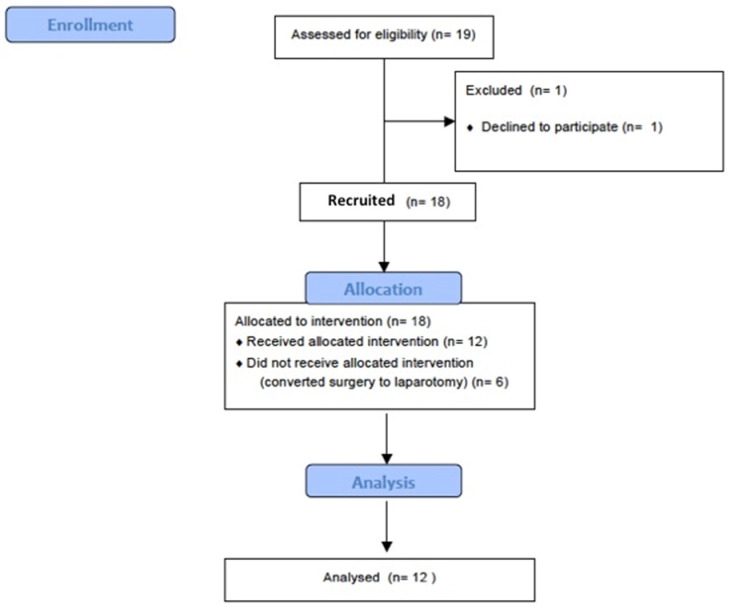
CONSORT flow diagram.

**Figure 2:**
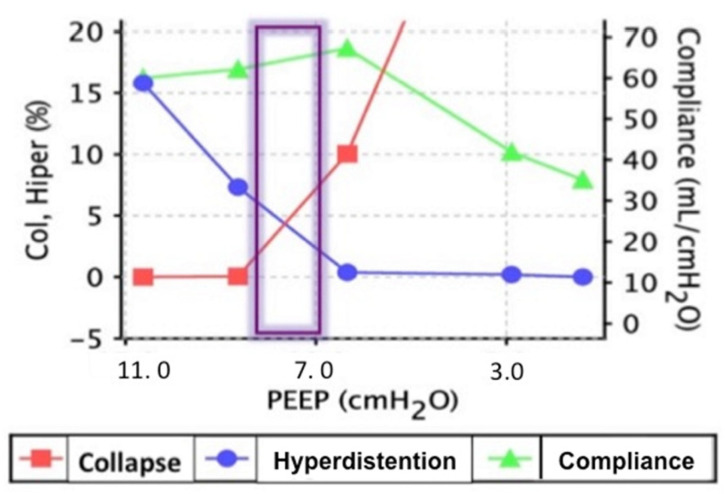
PEEP titration tool. The percentage of collapse and hyperdistention is shown on the left y axis and respiratory system compliance (Cs, mL/cmH_2_O) is shown on the right y axis; on the X axis, PEEP levels (cmH_2_O) are shown. Purple rectangle represents the “best PEEP” selected in order to minimize either collapse and hyperinflation, corresponding to the highest respiratory system static Compliance.

From 19 patients assessed for eligibility, 12 patients (83% female) were included in the study. The exclusion rate was 32% due to surgical conversion to laparotomy (Figure 1). The group of patients analyzed did not differ regarding the pre-surgical characteristics (median age in years, 68.5 (IQR 55–79); median BMI, 26.5 (IQR 19–30) kg/m^2^) and observation time (median duration of surgery was 195 (IQR 105–315) min), (Table 1). There were no significant differences in the hemodynamic parameters between the different study steps (Table 2). The mean PEEP applied after the recruitment maneuver was 7.91 ± 2.6 cmH_2_O (Table 2), and the PEEP titration tool (Figure 2) showed that this level represented the “best” compromise between the lower collapse and the lower hyperdistention and the highest Cs, meaning that moving away from the best PEEP level worsened the respiratory mechanics.

**Figure 3:**
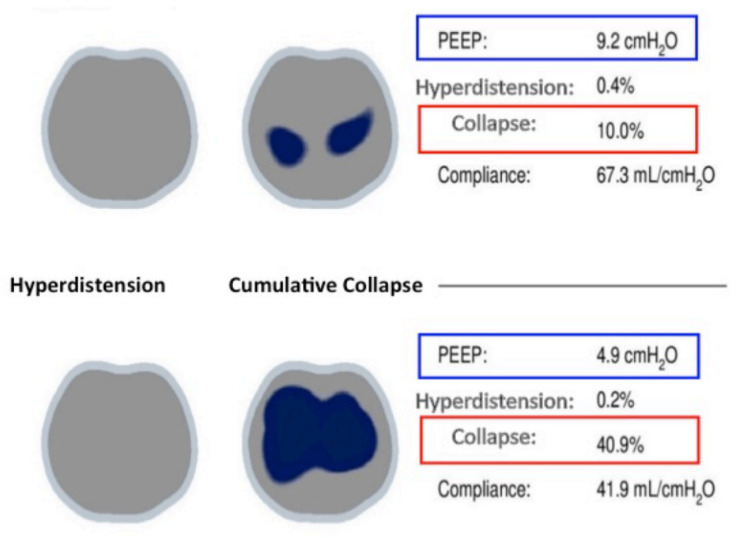
Ventilation distribution during a PEEP trial from one of the patients enrolled in this study, showing two levels of PEEP and the associated percentage of collapse, hyperdistention and corresponding respiratory system compliance (mL/cmH_2_O).

**Figure 4:**
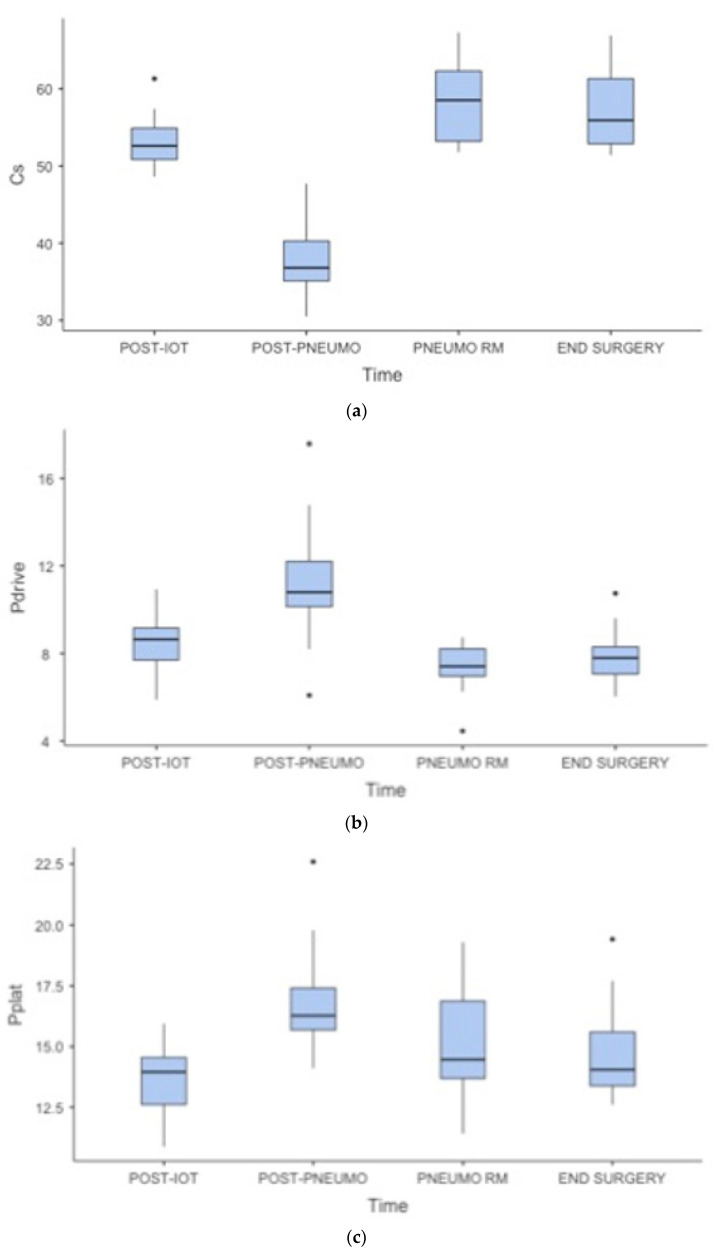
Respiratory parameters ((**a**) Cs, respiratory system compliance, mL/cmH_2_O; (**b**) Pdrive, driving pressure, cmH_2_O; (**c**) pplat, plateau pressure, cmH_2_O) throughout the four study steps (T1, after induction; T2, after pneumoperitoneum insufflation; T3, after a recruitment maneuver; T4, at the end of surgery after desufflation). * shows a *p* value < 0.05.

The random initial mean PEEP of 5 cmH_2_O applied after anesthesia induction and pneumoperitoneum insufflation was associated with 41% alveoli collapse and a lower Cs of 42 mL/cmH_2_O, whereas the application of a PEEP of 9 cmH_2_O was associated with a 10% collapse and an higher Cs value of 67.3 mL/cmH_2_O, as showed in Figure 3. Both PEEP levels were associated with a lower level of hyperdistention (0.2% and 0.4%, respectively). No significant changes were noted in mechanical ventilation parameters between the study steps (Table 2), except for Cs, which differed significantly between T2 and the other study times (Figure 4).

During the pneumoperitoneum insufflation period (T2), a decrease in Cs occurred, from 53.3 ± 3.54 to T1 to 38 ± 4.99 mL/cmH_2_O, followed by an increase to 58.4 ± 5.43 mL/cmH_2_O (*p* < 0.001) after the application of a selected value of 8 cmH_2_O of PEEP (T3). Pdrive followed the same behavior, with a significant, although still safe, increase from 8.5 ± 1.45 to 11.3 ± 2.96 cmH_2_O from T1 to T2, respectively (*p* < 0.001), (Figure 4). Both Cs and Pdrive remained stable at the end of surgery (T4). We observed a reduction in Pplat from 13.6 ± 1.51 to 17 ± 2.3 cmH_2_O from T1 to T2, and a subsequent reduction to 15.2 ± 2.36 cmH_2_O after the PEEP titration, although this was only slightly significant (*p* < 0.03), (Figure 4).

Throughout this study, higher oxygenation values could be observed after the application of PEEP (Table 2), with a PaO_2_ of 188 ± 66.7 mmHg at the end of surgery (T4, *p* < 0.001).

## 4. Discussion

The main findings of this study are as follows: (1) a selected-EIT guided external PEEP of 8 cmH_2_O is associated with improved respiratory mechanics, such as the driving pressure and static respiratory system compliance. (2) EIT application during laparoscopic abdominal surgery is confirmed as a feasible tool and helps with the avoidance of ventilation distribution impairment, resulting in better post-operative oxygenation.

Several studies have been published in which EIT was used to assess mechanical ventilation changes in intubated patients [18,19]. Bikker et al. [18] performed a PEEP decremental trial at four PEEP levels (15–10–5–0 cmH_2_O) in ICU patients. They found that tidal impedance increased only in the nondependent regions in patients with no lung disorders, after decreasing PEEP from 15 to 10 cmH_2_O. Another study published by the same authors demonstrated that the recruitment and overdistension of alveoli in response to different PEEP levels could be observed in the higher position on the thorax [19]. In our study, EIT electrodes were placed around the thorax at the level of the xiphoid process, as a good surrogate of global lung behavior [20], and due to its avoidance of imaging potential organ movements below the diaphragm [21].

Schaefer et al. [22] observed that EIT monitoring during abdominal surgery has significant limitations, due to the possible loss of impedance signal quality and the close proximity to the surgical site if the belt is positioned between the fifth and sixth intercostal spaces. However, atelectasis and ventilation shifts between dependent and nondependent lungs are more visible just above the diaphragm [23]. Nevertheless, other authors that conducted studies during abdominal laparoscopic surgery did not report the same technical issue, even though the belt was placed between the fifth and sixth intercostal spaces [17,24]. Moreover, the results of our study are not consistent with those of Erlandsson et al. [25], who found that PEEP of 10 cm H_2_O was not enough to prevent alveolar collapse after general anesthesia was induced in morbidly obese patients, since our population consisted of healthy adult patients, and thus a mean PEEP of 8 cmH_2_O was sufficient to counterbalance the effects of laparoscopy, recruit the lungs and restore a good level of oxygenation at the end of surgery. The parameters of lung mechanics may also be used to detect atelectasis and alveolar recruitment [16,26], and unlike EIT, which shows regional ventilation distribution in the monitored area, they are considered global parameters indicating changes in the whole lungs. Static respiratory system compliance (Cs) and plateau pressure (Pplat) in our examined group of patients showed typical changes described earlier in reports on respiratory mechanics during laparoscopy [27,28,29,30], with reduced compliance and increased plateau pressure during pneumoperitoneum insufflation and the reverse effect after the normalization of intra-abdominal pressure. Our group previously investigated the physiological effects of the open lung approach (OLA) in patients undergoing abdominal laparoscopic surgery, following the same approach applied in patients with ARDS consisting of a RM immediately followed by a standardized stepwise decremental PEEP trial, to find the “best PEEP”, i.e., the PEEP level that allows one to obtain the highest Cs during the decremental PEEP trial and that increases end-inspiratory transpulmonary pressure (PL), since we partitioned the respiratory system mechanics into their lung and chest-wall components by measuring esophageal pressure (Pes) as a surrogate for pleural pressure. Interestingly, the decremental PEEP trial targeting the highest Cs identified a PEEP level ranging from 6 to 10 cmH_2_O (mean: 8.9 ± 1.3 cmH_2_O) as the “best PEEP”, which is in line with the EIT’s “best PEEP” findings.

The novelty of our study is in the fact that we were able to visualize changes in driving pressure (Pdrive) step by step, and instead of using an esophageal balloon, we monitored and selected the “best” PEEP in a non-invasive manner, using EIT. During pneumoperitoneum insufflation, Cs decreased significantly, Pdrive increased significantly, whereas the increase in IAP did not impair oxygenation, which is in accordance with other studies [18]. EIT was also used to assess the modifying effects of a recruitment maneuver and PEEP on lung ventilation in patients undergoing general anesthesia for laparoscopy [17], showing that the application of 8 cmH_2_O of PEEP was able to reverse the ventilation distribution changes resulting from anesthesia induction and, at the same time, was high enough to prevent the adverse effects of the pneumoperitoneum insufflation [17]. Together with a limited tidal volume, the contemporary application of both PEEP and recruitment maneuvers is an indispensable part of a lung-protective mechanical ventilation strategy in patients undergoing abdominal surgery [31]. Our data showed that the recruitment maneuver accompanied by PEEP was able to prevent the effects of impaired ventilation observed in most laparoscopic surgical patients. Furthermore, the PEEP titration tool of TIMPEL was more accurate and helped to identify slight changes when decreasing PEEP in steps of 2 cmH_2_O.

In the course of PEEP titration, oxygenation in the PEEP-ventilated patients improved to some specific levels. A higher PaO_2_/FiO_2_ ratio could be observed at the PEEP level of 8 cmH_2_O, which is associated with better-distributed ventilation. In the pneumoperitoneum insufflation period, the PaO_2_/FiO_2_ ratio was slightly lower, although no significant difference was found. It seemed that EIT together with compliance and driving pressure were reasonable options in matching the ‘‘best’’ PEEP. Our results show that a non-invasive selection of 8 cmH_2_O of PEEP was not inferior to other invasive (previously descripted) methods to improve ventilation distribution and oxygenation—through recruiting dependent lungs and minimizing the overdistension of nondependent lungs—keeping Pdrive in a “safe” range in patients undergoing laparoscopic surgery, without significantly interfering with other respiratory or hemodynamic parameters. Notably, PEEP may also lead to negative effects with the reduction in cardiac output [17,31]. Unfortunately, we have no recorded data about this, so we have no further observations with respect to the potential for PEEP-related reduction in systemic blood flow during laparoscopic surgery. In spite of the raised IAP during PEEP ventilation, we did not observe any increase in MAP throughout our study (Table 2).

As is widely demonstrated, PPCs represent a large proportion of the risks related to major abdominal surgery and the intraoperative mechanical ventilation strategy, which may impair patient recovery and extend the length of stay in hospital [31]. Generally, most patients can be considered for laparoscopic surgery if they are able to tolerate general anesthesia. However, caution must be taken in the choice of laparoscopic surgery, because pneumoperitoneum insufflation may influence the pulmonary capacity or reserve for patients with severe pulmonary disease. This is probably the reason why we did not observe any potentially dangerous changes either in the respiratory mechanics parameters or oxygenation, since our group of patients were all ASA I-II with no impairment of respiratory function. We thus suggest that this type of monitoring might be beneficial particularly in other surgical branches (i.e., thoracic surgery), in order to closer monitor pulmonary function and avoid the onset of PPCs and delayed discharge from the hospital or increased admission to ICUs.

Our study has some limitations, such as the small sample size and no postoperative follow-up, which might have helped in stratifying the outcome of these patients. Future studies should improve these methodological shortcomings, with the real-time EIT machine being more available in the operating room theatre. This tool may help anesthesiologists to offer a better assessment and selection of the protective mechanical ventilation strategy.

## 5. Conclusions

In conclusion, to individualize the “best” PEEP level during abdominal laparoscopic surgery, EIT has been confirmed to be a feasible, safe, easy-to-use, non-invasive tool. The application of an initial recruitment maneuver under EIT monitoring, followed by a selected PEEP level, leads to (1) increased ventilation in the dependent lung areas, with significantly higher Cs and safer ranges of Pplat and Pdrive, (2) improved arterial oxygenation, and (3) negligible hemodynamic side effects.

## Figures and Tables

**Table 1:** Demographics.

Patient (n.)	Age (yrs)	Sex (M/F)	BMI (Kg/m^2^)	Comorbidities	ASA	Type of Surgery	Duration of Surgery (min)
1	72	M	25.9	DM II	II	Left hemicolectomy	105
2	79	F	28.1	HTN	II	Right hemicolecthomy	120
3	69	F	25.4	HTN	II	Left hemicolectomy	120
4	67	F	30.5	HTN, COPD	II	Ileo-cholic resection	250
5	68	F	23.3		I	Left hemicolectomy	315
6	73	F	29.3	HTN	II	Left hemicolectomy	180
7	69	F	23.9	HTN	II	Left hemicolectomy	180
8	77	F	26.6	HTN	II	Right Total colectomy	240
9	64	F	19.3	CHF	II	Left Total colectomy	270
10	55	F	27.7		I	Right hemicolecthomy	210
11	57	F	21.7		I	Left hemicolectomy	180
12	65	M	26.5	HTN	II	Left hemicolectomy	255

BMI: body mass index.

**Table 2:** Respiratory and hemodynamics parameters.

	T0 (Baseline)	T1 (IOT)	T2 (Pneumo)	T3 (Pneumo-RM)	T4 (End Surgery)	*p* Value
pH	7.38 ± 0.026	7.41 ± 0.06	7.37 ± 0.07	7.42 ± 0.05	7.35 ± 0.05	0.182
PaO_2_ mmHg	78.1 ± 9.49	130 ± 48.1	138 ± 42.5	149 ± 43.5	188 ± 66.7	<0.001
PaCO_2_ mmHg	39.1 ± 3.18	37.9 ± 4.44	42.4 ± 5.89	44.8 ± 5.19	40.3 ± 6.85	0.075
PaO_2_/FiO_2_	372 ± 45	313 ± 130	335 ± 116	367 ± 111	371 ± 80.4	0.819
PAM mmHg	95.8 ± 15	84.2 ± 12.7	84.2 ± 14.5	81.8 ± 13.5	78.5 ± 11.4	0.100
HR bpm	81 ± 11.6	75.8 ± 9.86	77 ± 9.40	72.3 ± 5.06	70.6 ± 8.18	0.279
Vt mL		452 ± 74.7	426 ± 104	425 ± 61.2	447 ± 69.5	0.747
Ve L/min		5.85 ± 0.71	5.5 ± 1.16	5.85 ± 0.96	5.96 ± 1.13	0.715
PEEPext cmH_2_O		5.08 ± 0.7	5.67 ± 1.23	7.91 ± 2.6	6.83 ± 2.5	0.027
Pplat cmH_2_O		13.6 ± 1.51	17 ± 2.3	15.2 ± 2.36	14.7 ± 2.07	0.003
Pdrive cmH_2_O		8.5 ± 1.45	11.3 ± 2.96	7.33 ± 1.21	7.87 ± 1.34	<0.001
Cs mL/cmH_2_O		53.3 ± 3.54	38 ± 4.99	58.4 ± 5.43	57 ± 5.10	<0.001
RR bpm		12.7	13	14.3	13.6	0.174

MAP, mean arterial pressure; HR, hearth rate; Vt, tidal volume; Ve, minute ventilation; PEEPext, external positive end-expiratory pressure; Pplat, plateau pressure; Pdrive, driving pressure; Cs respiratory system static compliance; RR, respiratory rate.

## Data Availability

Data supporting reported results can be request to the corresponding author.
